# Inhibition of CK2 Reduces NG2 Expression in Juvenile Angiofibroma

**DOI:** 10.3390/biomedicines10050966

**Published:** 2022-04-21

**Authors:** Anne S. Boewe, Silke Wemmert, Philipp Kulas, Bernhard Schick, Claudia Götz, Selina Wrublewsky, Mathias Montenarh, Michael D. Menger, Matthias W. Laschke, Emmanuel Ampofo

**Affiliations:** 1 Institute for Clinical & Experimental Surgery, Saarland University, 66421 Homburg, Germany; anne.boewe@uks.eu (A.S.B.); selina.wrublewsky@uks.eu (S.W.); michael.menger@uks.eu (M.D.M.); matthias.laschke@uks.eu (M.W.L.); 2 Department of Otolaryngology, Saarland University Medical Center, 66421 Homburg, Germany; silke.wemmert@uks.eu (S.W.); philipp.kulas@uks.eu (P.K.); bernhard.schick@uks.eu (B.S.); 3 Medical Biochemistry and Molecular Biology, Saarland University, 66421 Homburg, Germany; claudia.goetz@uks.eu (C.G.); mathias.montenarh@uks.eu (M.M.)

**Keywords:** juvenile angiofibroma, NG2, CK2, CX-4945, SGC-CK2-1, migration, proliferation

## Abstract

Juvenile angiofibroma (JA) is a rare fibrovascular neoplasm predominately found within the posterior nasal cavity of adolescent males. JA expresses the proteoglycan nerve–glial antigen (NG)2, which crucially determines the migratory capacity of distinct cancer cells. Moreover, it is known that the protein kinase CK2 regulates NG2 gene expression. Therefore, in the present study, we analyzed whether the inhibition of CK2 suppresses NG2-dependent JA cell proliferation and migration. For this purpose, we assessed the expression of NG2 and CK2 in patient-derived JA tissue samples, as well as in patient-derived JA cell cultures by Western blot, immunohistochemistry, flow cytometry and quantitative real-time PCR. The mitochondrial activity, proliferation and migratory capacity of the JA cells were determined by water-soluble tetrazolium (WST)-1, 5-bromo-2′-deoxyuridine (BrdU) and collagen sprouting assays. We found that NG2 and CK2 were expressed in both the JA tissue samples and cell cultures. The treatment of the JA cells with the two CK2 inhibitors, CX-4945 and SGC-CK2-1, significantly reduced NG2 gene and protein expression when compared to the vehicle-treated cells. In addition, the loss of CK2 activity suppressed the JA cell proliferation and migration. These findings indicate that the inhibition of CK2 may represent a promising therapeutic approach for the treatment of NG2-expressing JA.

## 1. Introduction

Juvenile angiofibroma (JA) is a pseudo-encapsulated mass, consisting of vascular and fibrous stromal tissue. Although it is a benign tumor, JA is capable of spreading through natural foraminas and fissures affecting the pterygopalatine and intratemporal fossa with erosion of the medial pterygoid lamina [1,2]. In addition, the extensive vascularization of this tumor and extension into the orbits and skull base may complicate surgical interventions due to a substantial risk of intraoperative bleeding [3,4].

In the last years, intensive research efforts have been made to find alternative strategies for the treatment of JA [5,6,7,8]. Targeting specific proteins that control JA growth and invasion may represent a particularly effective therapeutic approach [7,9]. In this context, we already showed a higher expression of the proteoglycan nerve glial (NG)2, also known as chondroitin sulfate proteoglycan 4 (CSPG4), in JA when compared to the control nasal mucosa tissue samples [10]. NG2 is a type-1 transmembrane protein. Its expression is restricted to distinct cell types, including pericytes, different progenitor cells and glioblastoma multiforme (GBM) cells [11]. The extracellular domain of the proteoglycan interacts with components of the extracellular matrix (ECM) [12], as well as with other membrane proteins, such as integrin β1, platelet-derived growth factor (PDGF)-α and fibroblast growth factor (FGF)2 [13]. These interactions trigger signaling transductions promoting cell proliferation and cell motility [14,15,16,17,18].

Of interest, we have recently identified the protein kinase CK2 as a novel regulator of NG2-dependent signaling pathways in pericytes and GBM [19,20]. This kinase is ubiquitously expressed and consists of two catalytic (CK2α or CK2α′) and two regulatory (CK2β) subunits [21]. CK2 has more than 500 substrates and, thus, is involved in various cellular processes, such as differentiation, cell cycle regulation and energy metabolism [22]. Moreover, the overexpression of CK2 promotes the development and progression of benign and malignant tumors [22,23,24,25]. Hence, several CK2 inhibitors have been developed as potential anti-cancer drugs, including CIGB-300 [26], SGC-CK2-1 [27] and CX-4945 [28].

In the present study, we analyzed whether CK2 inhibition reduces the cell proliferation and cell migration of JA by downregulating NG2 expression. To test this, we examined the effect of the two CK2 inhibitors, SGC-CK2-1 and CX-4945, on NG2 expression, mitochondrial activity, cell proliferation and cell migration in patient-derived JA cell cultures.

## 2. Materials and Methods

### 2.1. Antibodies

The anti-NG2 antibody (sc-166251) and the anti-CK2β antibody (E9) were from Santa Cruz Biotechnology (Heidelberg, Germany). The anti-α-tubulin antibody (66031) was from Proteintech Germany GmbH (St. Leon-Rot, Germany). The anti-AKT1/2/3 antibodies (11E7) and poly (ADP-ribose) polymerase (PARP) (9542) were from Cell Signaling (Frankfurt am Main, Germany). The anti-pAKT^S129^ antibody (ab133458) and the anti-NG2 antibody (ab129051) were from Abcam (Cambridge, UK). The anti-CD31 and anti-vimentin antibodies were from Dako Agilent (Hamburg, Germany). The anti-CK2α and anti-CK2β antibodies were generated, as described previously [29]. The peroxidase-labeled anti-rabbit antibody (NIF 824) and the peroxidase-labeled anti-mouse antibody (NIF 825) were from GE healthcare (Freiburg, Germany). The anti-chondroitin sulfate proteoglycan 4 (NG2; 562415) was from BD Biosciences (Heidelberg, Germany). The anti-5-bromo-2′-deoxyuridine (BrdU) antibody was from eBioscience (Thermo Fisher Scientific, Karlsruhe, Germany).

### 2.2. Patient-Derived JA Tissue Samples

Paraffin-embedded archived tissue samples of 5 male patients (age: 13–20 years) operated and diagnosed at Saarland University Medical Center between 2016–2018 were analyzed in this study. The tumor stage ranged from I1 to III3. Written informed consent was obtained from all the patients and the use of the human tissues was in accordance with the code of ethics of the World Medical Association (Declaration of Helsinki), as well as approved by the Institutional Review Board (#218/10) at Saarland University.

### 2.3. Cell Culture

The JA cell cultures were generated from at least 1 cm^3^ of native tumor samples, as described previously [30]. Briefly, the tissue samples were mechanically dissected and cultivated in DMEM F/12 Glutamax (Thermo Fisher Scientific, Darmstadt, Germany) supplemented with 10% fetal calf serum (FCS) (Thermo Fisher Scientific, Darmstadt, Germany), 1% penicillin–streptomycin, 1% sodium pyruvate, 10 µg/mL gentamycin (PAN-Biotech GmbH, Aidenbach, Germany) and 2.5 µg/mL amphotericin B (Thermo Fisher Scientific, Darmstadt, Germany) at 37 °C and 5% CO_2_. The medium was changed twice a week and the cells were passaged at 70% confluence by a split ratio of 1:3 with accutase solution (PromoCell, Heidelberg, Germany).

### 2.4. Immunohistology

For the preparation of immunohistological sections, the specimens of JA tissue were fixed for 24 h in 4% formalin. Thereafter, the sections were stained with the antibodies anti-CD31, anti-vimentin, anti-NG2, anti-CK2α and anti-CK2β. The DAKO Fast Red Kit (K5005, Agilent, Frankfurt, Germany) was used for detection according to the manufacturer’s instructions. Afterwards, the sections were counterstained with Mayer’s Hematoxylin (MHS32, Merck, Darmstadt, Germany). The quantification of the positively stained cells was performed per section by FIJI software (NIH, Bethesda, Maryland, USA) and is given as a % of all the cells per section.

For further immunohistochemical analyses, the sections were stained with anti-NG2 and anti-vimentin antibodies, which were detected by their corresponding fluorescence-coupled secondary antibodies. Hoechst 33342 was used to stain the cell nuclei (2 µg/mL). The sections were analyzed by means of fluorescence microscopy (BX61; Olympus, Hamburg, Germany).

### 2.5. Western Blot Analysis

The JA tissue samples, untreated JA cells and JA cells treated with CX-4945 (10 µM), SGC-CK2-1 (5 µM) or DMSO were lysed and the protein concentration was measured using a Bradford protein assay (Bio-Rad Laboratories, Feldkirchen, Germany). Bovine serum albumin (BSA) was used as standard. The JA whole cell extracts were separated through a 7.5% and 12% SDS-polyacrylamide gel and subsequently transferred onto a polyvinylidene difluoride (PVDF) membrane (Bio-Rad Laboratories, Feldkirchen, Germany). After blocking with 5% BSA in tris-buffered saline (TBS) (0.1% Tween20) for 1 h, the membrane was incubated with the primary antibodies against NG2, CK2α, CK2β, α-tubulin, pAKT^S129^ and AKT (dilution 1:100) in TBS (0.1% Tween20; 1% BSA) overnight followed by the corresponding secondary antibodies (1:1000). The protein expression was visualized by luminol-enhanced chemiluminescence (ECL) Western blotting substrate (Bio-Rad Laboratories, Feldkirchen, Germany) in an ECL ChemoCam Imager (Intas, Göttingen, Germany). The intensity of the measured signals was quantified using ImageJ software (NIH, Bethesda, MD, USA) and normalized to the loading control (α-tubulin).

### 2.6. CK2 Phosphorylation Assay

Cell extracts from the JA tissue samples were prepared for kinase filter assays, as previously described in detail [31]. The incorporation rate of [^32^P] phosphate into the CK2-specific substrate peptide with the sequence RRRDDDSDDD was measured. Twenty µL of kinase buffer (50 mM Tris/HCl, pH 7.5, 100 mM NaCl, 10 mM MgCl_2_, 1 mM DTT) containing 20 µg of proteins was mixed with 30 µL of CK2 mix (25 mM Tris/HCl, pH 8.5, 150 mM NaCl, 5 mM MgCl_2_, 50 µM ATP, 1 mM DTT, 0.19 mM substrate peptide) containing 10 µCi/500 µL [^32^P]yATP. The mixture was spotted onto a P81 ion exchange paper. The paper was washed three times with 85 mM H_3_PO_4_. After treatment with ethanol, the paper was dried and the Čerenkov radiation was determined in a scintillation counter.

### 2.7. Flow Cytometry

The untreated JA cells were washed with phosphate-buffered saline (PBS) and harvested with an enzyme-free dissociation buffer (Thermo Fisher Scientific, Darmstadt, Germany). The cells were incubated with a phycoerythrin (PE)-labeled primary anti-NG2 antibody for 30 min at room temperature. Thereafter, the cells were washed with PBS and the mean fluorescence intensity (MFI) of 1000 cells was analyzed by a FACSLyric™ flow cytometer (BD, Heidelberg, Germany). The non-expressing NG2 cell line HEK293 was used as a negative control.

To determine cell proliferation, the JA cells were treated with CX-4945 (10 µM), SGC-CK2-1 (5 µM) or DMSO and exposed to 10 µM BrdU for 48 h. Thereafter, the cells were fixed, permeabilized and incubated with an anti-BrdU antibody. The cells were then washed with PBS and the MFI of 500 cells was analyzed by a FACSLyric™ flow cytometer (BD, Heidelberg, Germany).

### 2.8. Water-Soluble Tetrazolium (WST)-1 Assay

A WST-1 assay (Roche, Mannheim, Germany) was used to analyze the effect of the inhibitors CX-4945 (ActivateScientific, Prien, Germany) and SGC-CK2-1 (Merk, Darmstadt, Germany) on the mitochondrial activity of the JA cells. Briefly, the cells were seeded in a 96-well culture plate at a density of 2 × 10^3^ cells/well. Thereafter, they were treated with 1 µM, 5 µM and 10 µM of CX-4945 and SGC-CK2-1 or the equivalent concentrations of the vehicle (DMSO). After 24 h and 48 h, 10 μL of WST-1 reagent was added into each well and the absorbance was measured at 450 nm in a microplate photometer.

### 2.9. Quantitative Real-Time PCR (qRT-PCR)

Total RNA was extracted with a QIAzol lysis reagent and transcribed into cDNA by using qScriber (highQu, Kraichtal, Germany) according to the manufacturer’s protocol. Briefly, the qRT-PCR analysis was performed by using ORA™ SEE qPCR Green ROX L Mix (highQu, Kraichtal, Germany). Forward and reverse primers (NG2 forward 5′-GCAAGCCGATGTGGATTC-3′ and reverse 5′-ATGGCGGATGGTAGGATG-3′; Glyceraldehyde-3-phosphate dehydrogenase (GAPDH) forward 5′-CCACCCATGGCAAATTCC-3′ and reverse 5′-ACTCCACGACGTACTCAG-3′) were used at a concentration of 500 nM. GAPDH was used as an endogenous control. Data collection was performed by means of a CFX96™ real-time system (Bio-Rad Laboratories, Feldkirchen, Germany) and the 2^−ΔΔct^ method.

### 2.10. Spheroid Sprouting Assay

The migratory capacity of the JA cells was determined by means of a sprouting assay. For this, 500 cells per well were seeded into 96-well plates coated with 100 µL 1% agarose to form spheroids. After 24 h, 30 spheroids were collected, resuspended in a collagen solution and transferred into a well of a 24-well plate. DMEM was added to the wells (0 h) and the spheroids were incubated for 48 h in the presence of DMSO, CX-4945 (10 µM) and SGC-CK2-1 (5 µM) at 37 °C and 5% CO_2_. The sprouting capacity was visualized by bright field microscopy. The sprouting area was assessed by means of the ImageJ software (NIH, Bethesda, Maryland, USA).

### 2.11. Statistical Analysis

After testing the data for normal distribution and equal variance, the differences between the two groups were assessed by the unpaired Student’s *t*-test. Statistics were performed by GraphPad Prism (Prism 8 software, GraphPad, San Diego, CA, USA). All the values are expressed as the mean ± SD. Statistical significance was accepted for *p* < 0.05.

## 3. Results

### 3.1. Expression of CK2 and NG2 in Patient-Derived JA Tissue Samples

**Figure 1:**
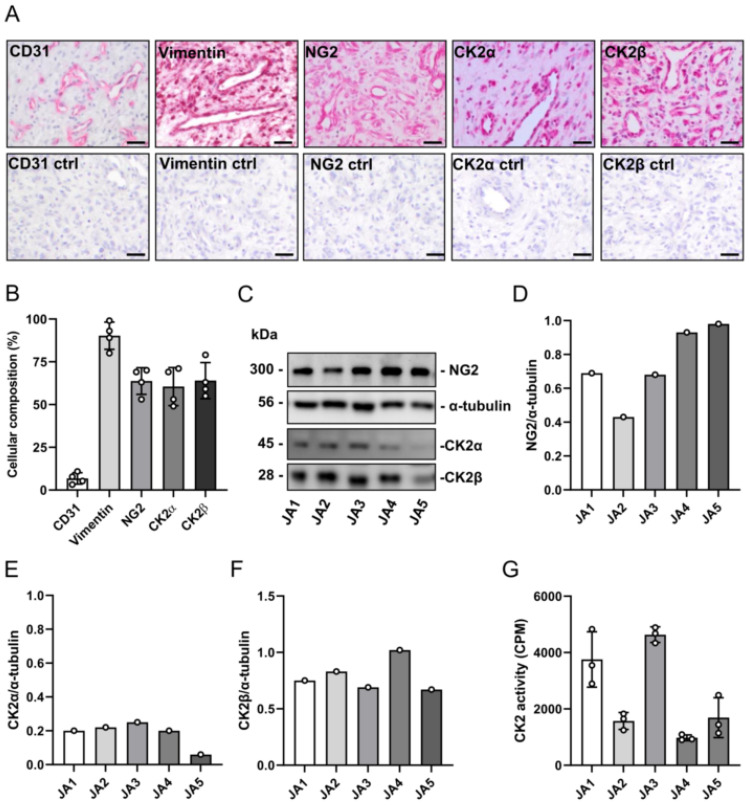
Expression of CK2 and NG2 in the patient-derived JA tissue samples. (**A**) Representative immunohistochemical stainings of CD31, vimentin, NG2, CK2α and CK2β from JA1 (upper panel). Scale bars: 25 µm. Immunohistochemical staining without primary antibodies served as a negative control (ctrl, lower panel). Scale bars: 25 µm. (**B**) Quantitative analysis of (**A**). The number of CD31-, vimentin-, NG2-, CK2α- and CK2β-positive cells is given as % of all the cells per section. Mean ± SEM (*n* = 4). (**C**) JA tissue samples from 5 patients (JA1–JA5) were lysed and the expression of NG2, CK2α, CK2β and α-tubulin (as a loading control) was analyzed by Western blot. (**D**–**F**) Quantitative analysis of (**C**). Data are expressed as the relative density ratio of NG2/α-tubulin (**D**), CK2α/α-tubulin (**E**) and CK2β/α-tubulin. (**G**) CK2 kinase activity (CPM) was measured by a CK2 kinase assay. Mean ± SD (*n* = 3).

JA consists of vascular and fibrous stromal tissue [1,2]. To assess whether the expression of CK2 and NG2 is restricted to distinct cell types, we first performed immunohistochemical stainings of a patient-derived JA tissue sample (JA1) (Figure 1A). CD31 and vimentin were stained to visualize the vascular and fibrotic areas, respectively (Figure 1A). We detected NG2 expression in the cells surrounding the blood vessels, as well as in the fibrotic tissue areas (Figure 1A). Similar expression patterns could be found for the CK2 subunits CK2α and CK2β (Figure 1A). We additionally determined the number of CD31-, vimentin-, NG2-, CK2α- and CK2β-positive cells within the tissue sample (Figure 1B). As expected, ~10% of the cells were positive for CD31 and ~90% of the cells stained positive for vimentin. The number of NG2-, CK2α- and CK2β-positive cells was ~60% (Figure 1B).

Moreover, we investigated the expression of CK2 and NG2 in five patient-derived JA tissue samples (JA1–JA5). All the tissue samples expressed the catalytic CK2α subunit, the regulatory CK2β subunits and the surface protein NG2 (Figure 1C–F). We additionally analyzed CK2 kinase activity, which was detected in all the patient-derived tissue samples (Figure 1G). Of note, the expression pattern and activity of CK2α and CK2β markedly differed between the individual tissue samples.

### 3.2. Expression of CK2 and NG2 in Patient-Derived JA Cells

**Figure 2:**
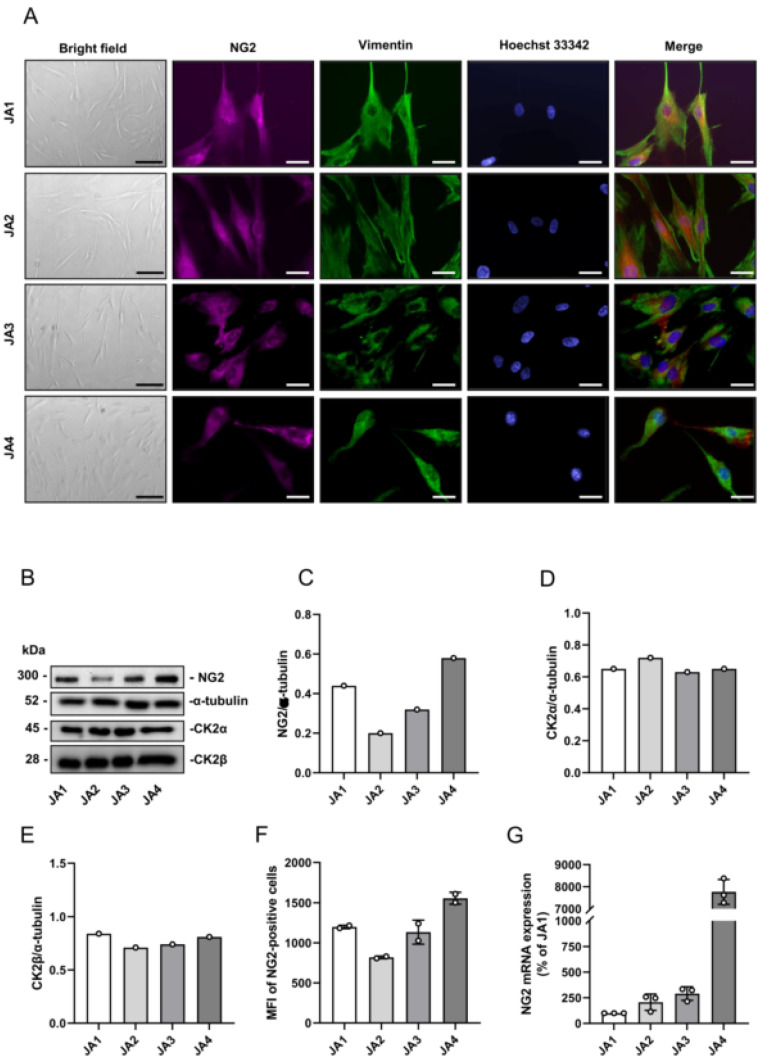
Expression of CK2 and NG2 in the patient-derived JA cells. (**A**) Bright field images (scale bars: 20 µm) and immunofluorescence stainings (scale bars: 50 µm) of NG2 (red), vimentin (green) and cell nuclei (blue) in the JA1–JA4 cells. (**B**) The JA1–JA4 cells were lysed and the expression of NG2, CK2α, CK2β and α-tubulin (as a loading control) was analyzed by Western blot. (**C**–**E**) Quantitative analysis of (**B**). Data are expressed as the relative density ratio of NG2/α-tubulin (**D**), CK2α/α-tubulin (**E**) and CK2β/α-tubulin. (**F**) NG2 surface expression of the JA1–JA4 cells was detected by flow cytometry. Mean ± SD (*n* = 2). (**G**) RNA was extracted from the JA1–JA4 cells and the gene expression of NG2 was examined by qRT-PCR (% of JA1). Mean ± SD (*n* = 3).

To study the influence of CK2 inhibition on NG2 expression in vitro, we additionally established cell cultures from the patient-derived JA1–JA4 tissue samples. The cells exhibited a fibroblast-like, multipolar shape in bright-field microscopy (Figure 2A). Immunofluorescence stainings demonstrated that the cells were positive for NG2 and for the intermediate filament protein vimentin (Figure 2A). Further Western blot analyses revealed that the cells from JA1–JA4 express NG2 as well as CK2α and CK2β (Figure 2B–E). We additionally verified NG2 protein expression in all the tissue samples by flow cytometry and NG2 gene expression by qRT-PCR analyses (Figure 2F,G).

### 3.3. CK2 Inhibition Suppresses the Proliferation of Patient-Derived JA Cells

**Figure 3:**
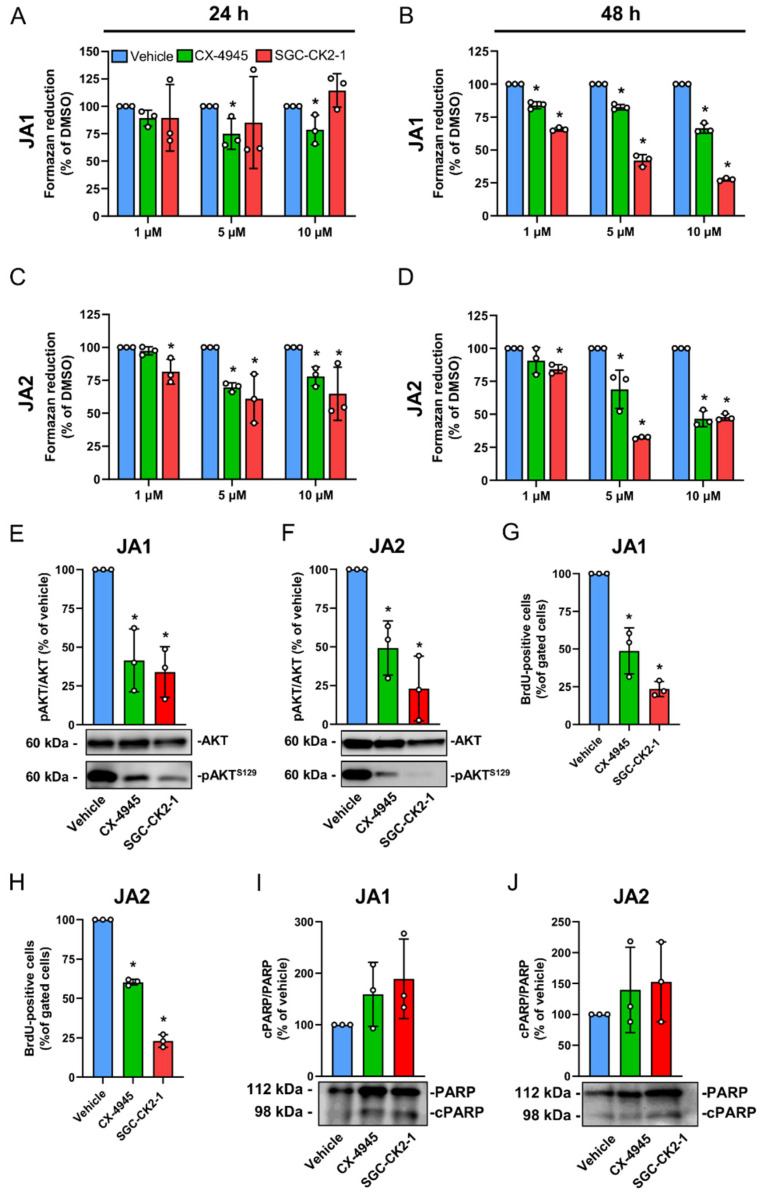
CK2 inhibition suppresses the proliferation of the JA cells. (**A**–**D**) The JA1 and JA2 cells were treated with the indicated concentrations of CX-4945 and SGC-CK2-1. The mitochondrial activity was measured by a WST-1 assay after 24 h and 48 h. Vehicle-treated cells served as controls and were set at 100%. Mean ± SD (*n* = 3). * *p* < 0.05 vs. vehicle. (**E**,**F**) The JA1 and JA2 cells were treated with CX-4945 (10 μM) and SGC-CK2-1 (5 μM) for 48 h. Vehicle-treated cells served as controls. The cells were lysed and the expression of AKT and pAKT^S129^ was assessed by Western blot. The relative density ratio of pAKT^S129^/AKT was determined and the vehicle-treated cells were set at 100%. Mean ± SD (*n* = 3). * *p* < 0.05 vs. vehicle. (**G**,**H**) JA1 and JA2 were treated as described in (**E**,**F**) and the incorporation of BrdU was analyzed by flow cytometry. The BrdU-positive cells were expressed to all the gated cells and the vehicle-treated cells were set at 100%. Mean ± SD (*n* = 3). * *p* < 0.05 vs. vehicle. (**I**,**J**) The JA1 and JA2 cells were treated with CX-4945 (10 μM) and SGC-CK2-1 (5 μM) for 48 h. Vehicle-treated cells served as controls. The cells were lysed and the expression of AKT and pAKT^S129^ was assessed by Western blot. The relative density ratio of cPARP/PARP was determined and the vehicle-treated cells were set at 100%. Mean ± SD (*n* = 3).

The effect of CK2 inhibition on NG2 expression was investigated in the two cell cultures JA1 and JA2. For this purpose, the cells were cultured for 24 h or 48 h with increasing concentrations of the CK2 inhibitors CX-4945 and SGC-CK2-1. Subsequently, the mitochondrial activity of the cells was assessed by means of a WST-1 assay (Figure 3A–D). We found that only 5 µM and 10 µM of CX-4945 attenuate the activity of the JA1 cells after 24 h when compared to the vehicle (Figure 3A). After a longer period of 48 h, both compounds significantly decreased the formazan reduction in a dose-dependent manner (Figure 3B). In contrast, treatment with CX-4945 or SGC-CK2-1 reduced the mitochondrial activity of the JA2 cells after 24 h, which was even more pronounced after 48 h (Figure 3C,D). Of note, the lowest activity after CK2 inhibition was detected in the cells treated with 10 µM CX-4945 or 5 µM SGC-CK2-1 for 48 h. Therefore, we decided to use these concentrations over 48 h to study the effect of CK2 inhibition on cell proliferation and apoptosis. First, we analyzed whether both compounds effectively inhibited the CK2 activity in the JA1 and JA2 cells. Our Western blot analyses revealed an attenuated AKT^S129^ phosphorylation after exposure of the cells to CX-4945 and SGC-CK2-1 (Figure 3E,F). As expected, the two inhibitors significantly reduced cell proliferation, as shown by a lower number of BrdU-positive cells (Figure 3G,H). Moreover, the Western blot analyses revealed an increased, but not significantly higher, protein level of cleaved PARP (Figure 3I,J).

### 3.4. CK2 Inhibition Reduces NG2 Expression and the Migratory Capacity of Patient-Derived JA Cells

**Figure 4:**
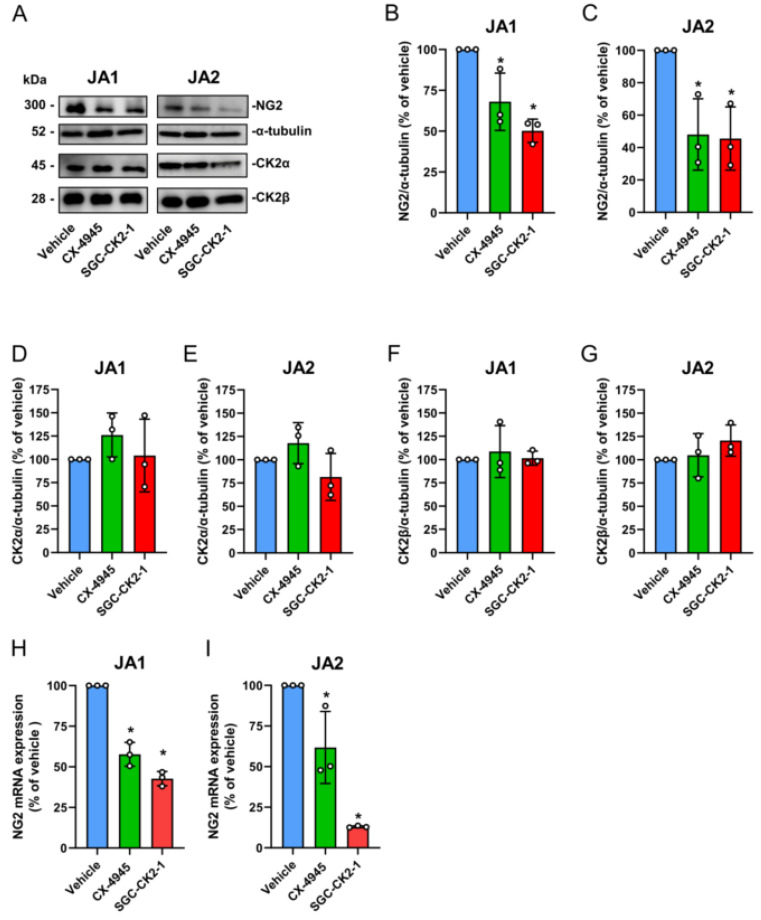
CK2 inhibition reduces the NG2 expression of the JA cells. (**A**–**D**) The JA1 and JA2 cells were treated with CX-4945 (10 μM) and SGC-CK2-1 (5 μM) for 48 h. Vehicle-treated cells served as controls. The cells were lysed and the expression of NG2, CK2α, CK2β and α-tubulin (as a loading control) was assessed by Western blot. The relative density ratio of NG2/α-tubulin (**B**,**C**), CK2α/α-tubulin (**D**,**E**) and CK2β/α-tubulin (**F**,**G**) was determined and the vehicle-treated cells were set at 100%. Mean ± SD (*n* = 3). * *p* < 0.05 vs. vehicle. (**H**,**I**). The gene expression of NG2 was measured by qRT-PCR. Vehicle-treated cells were set at 100%. Mean ± SD (*n* = 3). * *p* < 0.05 vs. vehicle.

We next analyzed the effect of CX-4945 and SGC-CK2-1 on NG2 expression. We detected a markedly decreased expression of NG2 in the JA1 and JA2 cells (Figure 4A–C). In contrast, the expression of CK2α and CK2β was not affected by CX-4945 or SGC-CK2-1 treatment (Figure 4A,D–G). Additional analyses of the NG2 gene expression in these cells revealed that the loss of CK2 activity significantly attenuates NG2 mRNA levels (Figure 4H,I).

**Figure 5:**
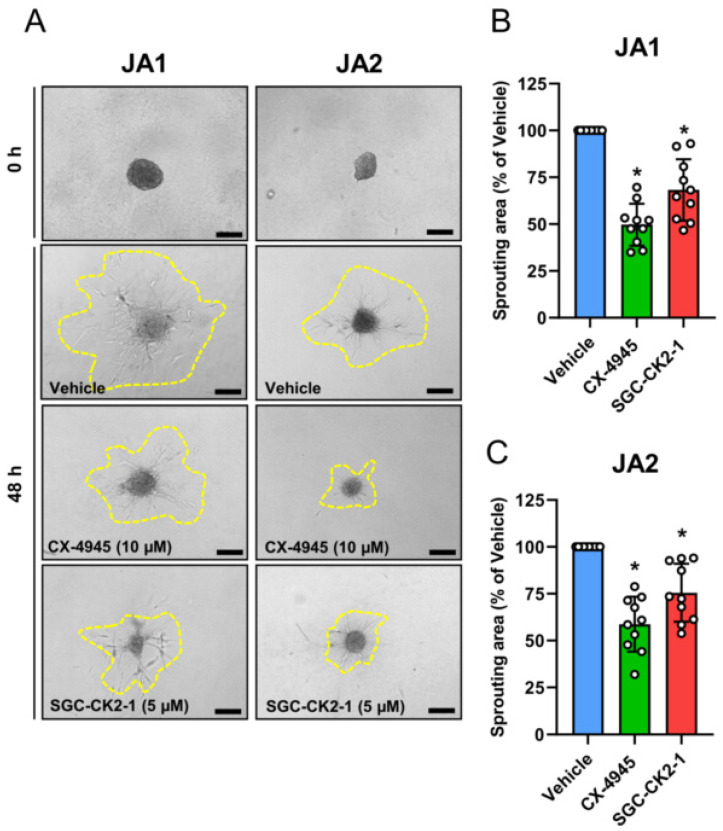
CK2 inhibition reduces the migratory activity of the patient-derived JA cells. (**A**) The JA1 and JA2 cells were cultivated by means of the liquid overlay technique for 24 h and the resulting spheroids were embedded into a collagen matrix. Bright field images of the sprouting area around the spheroids (the border marked by a broken yellow line) were taken immediately after embedding (0 h) and 48 h after treatment with the vehicle, CX-4945 (10 µM) and SGC-CK2-1 (5 µM). Scale bars: 100 µm. (**B**,**C**) The sprouting area was assessed 48 h after the treatment of the JA1 (**B**) and JA2 (**C**) spheroids with the vehicle, CX-4945 (10 µM) and SGC-CK2-1 (5 µM). Data are expressed as a % of the vehicle-treated spheroids. Mean ± SD (*n* = 10). * *p* < 0.05 vs. vehicle.

The binding of NG2 to proteins of the ECM triggers cell migration [12,32,33]. To analyze the influence of CK2 on NG2-dependent JA cell migration, we finally performed a collagen sprouting assay. For this purpose, 500 JA1 or JA2 cells were cultivated by using the liquid overlay technique to generate spheroids, which were subsequently embedded within a collagen matrix. The sprouting capacity of the cells was assessed over 48 h. We found that the cells of the vehicle-treated JA1 and JA2 spheroids rapidly grew out. This outgrowth was more pronounced in the JA1 spheroids compared to the JA2 spheroids (Figure 5A). Notably, in both groups, CK2 inhibition with CX-4945 or SGC-CK2-1 significantly reduced the sprouting capacity of the spheroids (Figure 5B,C).

## 4. Discussion

In the present study, we demonstrated that the pharmacological inhibition of CK2 by SGC-CK2-1 and CX-4945 significantly reduces NG2 gene expression in the patient-derived JA cells. Moreover, we detected a markedly decreased proliferation and migration in the JA cells after CK2 inhibition. JA are pseudo-encapsulated, heterogeneous benign tumors consisting of abundant vascular channels lacking the normal muscular layer in the channel wall and a network of fibrocollagenous tissue [34]. We already showed that NG2 mRNA expression is upregulated in JA when compared to the control nasal mucosa tissues [10]. This indicates that this proteoglycan may play an important role in JA cell growth and migration. In contrast, the expression pattern of CK2 in JA has not been analyzed so far. Of interest, our results demonstrate for the first time that NG2, as well as the CK2 subunits CK2α and CK2β, are expressed in all the patient-derived JA tissue samples.

During the last years, several studies reported that the selective inhibition of CK2 induces apoptosis in tumor cells relative to normal cells [35]. Accordingly, many CK2 inhibitors have been generated as anti-cancer drugs [28,36,37,38,39]. For instance, CX-4945 (IC50 = 360 nM) is currently being tested in phase I and II clinical trials for the treatment of various cancer types, including medulloblastoma (NCT03904862), cholangiocarcinoma (NCT02128282) and multiple myeloma (NCT01199718). Recently, Wells et al. [27] suggested SGC-CK2-1 (IC50 = 36 nM) as an efficient CK2 inhibitor [27]. This molecule is considered as non-toxic and, thus, has been proposed to be suitable not only for the treatment of cancers, but also for other pathological conditions, such as neurodegenerative diseases [27,40].

To study the effects of CX-4945 and SGC-CK2-1 on NG2 expression in JA, we first established cell cultures from the JA tissue samples, which is quite challenging due to the scarcity and the heterogeneity of this tumor type [10]. We showed that the JA cells expressed both NG2 and CK2. We next determined suitable concentrations that efficiently suppress CK2 activity. The exposure of the JA cells to 10 µM of CX-4945 and 5 µM of SGC-CK2-1 reduced the phosphorylation of AKT at serine 129, a specific CK2 phosphorylation site [41]. More importantly, both inhibitors significantly reduced the gene expression of NG2. This is in line with our previous studies showing that the loss of CK2 activity decreases NG2 gene expression in human placenta pericytes and in human NG2-positive GBM cells due to the inhibitory action of the transcription factor Sp1 [19,20,42,43]. Sp1 regulates the expression of multiple genes and its overexpression contributes to the malignant phenotype of a variety of human cancers by upregulating genes that enhance proliferation and migration [44]. The expression of Sp1 in JA has not been investigated so far. Hence, we can only assume that CK2 also regulates the expression of NG2 via Sp1 in JA.

NG2 promotes cell migration via its binding to the ECM [12,32,33]. Hence, it is conceivable that a diminished NG2 expression caused by CK2 inhibition results in a markedly decreased cell migration. In fact, our collagen matrix-based spheroid sprouting assays revealed that treatment with CX-4945 and SGC-CK2-1 significantly reduces the sprouting capacity of the JA cells. Notably, JA contains a higher amount of collagen type VI when compared to the control nasal mucosa [10]. Moreover, it is known that the specific binding of NG2 to collagen type VI triggers cell migration by convergent signal transduction pathways [45]. These findings indicate that the inhibition of CK2 and the resulting downregulation of NG2 expression may be highly effective for the selective treatment of JA.

In the last decade, several studies described the involvement of specific genes and signaling pathways, such as Wnt, in JA progression [46]. In particular, Wnt5B, a member of the Wnt family, is overexpressed in JA [47]. Of note, CK2 acts as a positive regulator of Wnt signaling [48] and several studies have already suggested that pharmacological CK2 inhibition may be a promising therapeutic approach for the treatment of tumors with high Wnt activity, such as chronic lymphocytic leukemia and B-cell lymphomas [49,50]. Accordingly, it may be speculated that besides the downregulation of NG2, the suppression of Wnt signaling by CK2 inhibition also contributed to the reduced migratory activity of the JA cells in our spheroid sprouting assays.

Besides the effect of NG2 on cell migration, the proteoglycan also promotes cancer growth by altering the expression of genes regulating cell proliferation and apoptosis [32,51]. Accordingly, the inhibition of NG2-dependent pathways is thought to be a promising therapeutic approach for the treatment of NG2-positive tumors [52]. In the present study, we detected a significantly reduced cell proliferation of and a slightly increased induction of apoptosis in the JA cells after CK2 inhibition, which may be caused by the reduced NG2 expression. However, it should be noted that CK2 regulates cell cycle progression and apoptosis via the phosphorylation of different proteins [53,54,55,56,57]. Therefore, further detailed analyses are required to study the effects of the reduced NG2 expression after CK2 inhibition on these processes.

Treatment with the cytotoxic CK2 inhibitors 4,5,6,7-tetrabromobenzotriazole (TBB), 2-dimethylamino-4,5,6,7-tetrabromo-1H-benzimidazole (DMAT) and CX-4945 typically leads to massive necrotic and apoptotic cell death [58]. Although the effectiveness and benefit of these compounds have been acknowledged in numerous cancer studies, they have also been recognized as hazardous substances due to their potential mutagenic, carcinogenic and reproductive toxicity properties [28,38,58,59,60,61]. In contrast, the novel inhibitor SGC-CK2-1 is not only highly specific for CK2, but also non-toxic [27]. Considering our novel finding that the inhibition of CK2 reduces NG2 expression and NG2-dependent cell migration in JA, SGC-CK2-1 may, thus, be a promising candidate for the future treatment of this benign tumor type, which is found particularly in young patients.

## Data Availability

Data are contained within the article.
